# U.S. healthcare provider risk perceptions of tobacco and nicotine-containing products

**DOI:** 10.1007/s11739-026-04321-1

**Published:** 2026-04-13

**Authors:** Susan Martelle, Deena Battista, Michael Polster

**Affiliations:** 1 Survey Research, RAI Services Company, Winston-Salem, USA; 2 Womble Bond Dickinson, Winston-Salem, USA; 3 Naxion, 1835 Market St, Philadelphia, PA 19073 USA

**Keywords:** HCP, Risk perceptions, Tobacco products, Nicotine

## Abstract

U.S. healthcare providers (HCPs) are a valuable source of information about the health impact of using tobacco products and the health benefits of quitting cigarette smoking thus it is important to understand their perceptions of the absolute and relative risks of tobacco and nicotine-containing products (TNPs). The study objectives were to assess: (a) HCP perceptions about the relative risks of TNPs; (b) how much of the risk of smoking cigarettes HCPs attribute to combustion versus other factors; and (c) whether HCPs believe that partial or complete switching from cigarettes to using other TNPs can reduce health risks. A 15 min self-administered online survey was completed by 700 HCPs enrolled in a national online panel. Mean overall health risk ratings on a 100-point scale differ significantly for cigarettes (95.9), e-cigarettes (79.5), smokeless tobacco (76.4), nicotine pouches (60.3), and nicotine replacement therapy (40.3). HCPs attribute less than 40% of the risk of lung cancer associated with smoking cigarettes to combustion. Half of HCPs do not believe that switching completely from cigarettes to e-cigarettes or smokeless tobacco can reduce health risks. These findings indicate that HCPs are aware that not all TNPs present the same health risks, but that their perceptions of the relative risks of combustible and non-combustible products are not consistent with published estimates from authoritative sources. HCPs are generally unaware that the smoke from burning tobacco is the primary source of risk associated with cigarette smoking and most do not believe that switching completely from cigarettes to e-cigarettes or smokeless tobacco products can reduce health risks. Together, these findings suggest that many HCPs do not have accurate information about the relative risks of non-combustible TNPs and that broadening awareness about the harm reduction associated with those products is a vital step to ensuring that HCPs can provide their patients who smoke with accurate information about the benefit of switching completely from cigarettes to other types of non-combustible TNPs.

**Supplementary Information:**

The online version contains supplementary material available at 10.1007/s11739-026-04321-1.

## Introduction

The introduction of the Family Smoking Prevention and Tobacco Control Act (FSPTCA) granted the Food and Drug Administration (FDA) regulatory oversight of tobacco products in 2009. Since the passage of that legislation, there have been substantial changes in the U.S. tobacco market, most notably, FDA authorization of new, non-combustible tobacco and nicotine-containing products (TNPs) including e-cigarettes and nicotine pouches. Those new products have been deemed to be appropriate for the protection of the public health largely because the benefits to smokers who switch completely to a lower risk TNP outweigh the risks to the population as a whole [1]. Despite substantial evidence that non-combustible TNPs are associated with less risk than cigarettes [2, 3] the public remains markedly unaware of the relative risks of these products. For example, only about 20% of both smokers and non-smokers believe that e-cigarettes contain fewer harmful chemicals than cigarettes [4], and less than 15% of U.S. adults believe that e-cigarettes are less harmful than cigarettes [5].

As a trusted source of medical information for many adults, healthcare providers (HCPs) are in an ideal position to educate patients who smoke cigarettes about the continuum of risk of TNPs. The accuracy of the information and the degree of counseling competence, however, depends on their level of evidence-based knowledge of the relative risks of those products, which includes knowing that non-combustible TNPs are associated with less risk than cigarettes, that inhaling smoke from burning tobacco (combustion) is the primary source of risk associated with smoking cigarettes [6], and that switching from cigarettes to a non-combustible product can reduce the health risk from cigarette smoking [2]. Yet, the limited literature on HCP perceptions and beliefs suggests that many HCPs do not have an accurate understanding of the relative risks of TNPs. For example, about 80% of physicians incorrectly believe that nicotine contributes to chronic obstructive pulmonary disease (COPD) and cancer [7], and 60% of physicians believe that all forms of tobacco are equally harmful [8]. Notably, in a speech at the Food and Drug Law Institute’s (FDLI) Tobacco and Nicotine Products Regulation and Policy Conference in October 2025, the Director of FDA’s Center for Tobacco Products (CTP) referenced HCPs not being adequately informed about the relative risk of various categories of tobacco products and that some of them mistakenly believe that nicotine is a carcinogen [9].

The current study was designed to examine HCP perceptions and beliefs on TNPs, including the extent of the health risk they associate with different products, awareness of the source of risk from smoking cigarettes, and whether they believe switching from cigarettes to non-combustible TNPs reduces health risks. Whereas previous studies have focused on physicians, the present study also included nurse practitioners (NPs) and physician assistants (PAs) as they have patient-facing roles that often involve screening for tobacco use and discussing the benefits of quitting smoking with those patients who smoke cigarettes.

## Methods

### Sample

HCPs from Survey Healthcare Global’s national online panel were invited to participate in an online survey via an email invitation during the first week of February 2024. Sampled panel members who elected to participate answered a few qualifying questions (*i.e.*, type of medical license held, primary medical specialty, state where HCP primarily practiced medicine, and amount of time spent in direct patient care per week). The sample included a total of 700 HCPs, all of whom spend at least 50% of their time in direct patient care, with 100 respondents in each of the following specialties to allow for between-specialty comparisons: family practitioners/general practitioners (FPs/GPs), internal medicine (IM), obstetricians/gynecologists (OBGYN), cardiologists (CARD), pulmonologists (PULM), nurse practitioners/physician assistants (NPs/PAs) in primary care practices (FP/GP, IM), and NPs/PAs in specialty practices (OBGYN, CARD, PULM). OBGYNs were included because they have conversations with pregnant women about the risk that tobacco products pose to fetuses, and CARDS and PULMs were included because they treat many patients with smoking-related diseases (*e.g.*, hypertension, COPD).

### Procedures

Respondents completed a 15 min online survey that assessed HCP perceptions and behaviors related to TNPs (*see* Supplemental Materials for survey questions)*.* For the current analysis, the questions of interest were: the overall health-related risks (on a 100-point scale where “0” means “*No Risk*” and “100” means “*Substantial Risk*”) associated with the use of cigarettes, e-cigarettes, smokeless tobacco, nicotine pouches, and nicotine-replacement therapy (NRT); whether a person who replaces half or all of their cigarettes per day by using a non-combustible TNP, NRT, or no aid would reduce their health risks from smoking cigarettes; and the proportion of the risk of lung cancer associated with smoking cigarettes that is attributable to (a) smoke from burned tobacco, (b) nicotine, (c) other chemicals found in cigarettes, and/or (d) some other source. Sensitivity regarding misperception of the role of nicotine in cancer [8], led to an interim data analysis after approximately half of the data (n = 384) had been collected. The results of that analysis led to the word “inhaling” being removed from the first three response options to assess if it was influencing results. Although the change yielded statistically significant differences in the percent of risk attributable to smoke from burned tobacco (*t*_698 df_ = 2.52, *p* = 0.012) and nicotine (*t*_698 df_ = 2.73, *p* = 0.006), the effect sizes were both small (*d* = 0.25, and *d* = 0.27, respectively); thus, aggregated data (including both sets of response options) are presented (*see* Supplemental Table 1). Demographic variables (age, gender, practice location, years in practice, and most recent smoking cessation training) and personal TNP use history were also collected.

### Weighting and data analysis

To create a weighted total for the sample that represents the universe of HCPs in the specialties surveyed, the American Medical Association count of the specialty (corrected by prevalence based on eligibility) was divided by the sample size of that specialty. Separate weighted totals were also created for the two groups of NPs/PAs and the five medical specialties (*see* Supplemental Table 2 for details).

All data processing, analyses, and weighting were performed using Base SAS® 9.4 [10] with SAS/STAT 15.3 [11]. Standard errors for weighted statistics were calculated using Taylor Series Linearization in standard SAS procedures (*e.g.*, PROC SURVEYMEAN, PROC SURVEYREG) [11], assuming a stratified random sample design (using HCP specialty as the strata) along with the survey weights. For rating variables, weighted means and 95% confidence intervals (CIs) are provided. To calculate the perceived risk reduction (RR) associated with using each product relative to smoking cigarettes, each respondent’s risk rating for each product was subtracted from their risk rating for cigarettes and divided by their risk rating for cigarettes. For example:

$$RR_{e - cig} = \, \left( {Rating_{cig} {-} \, Rating_{e - cig} } \right) \, / \, Rating_{cig}$$

$$0.2 \, = \, \left( {100 \, {-} \, 80} \right) \, / \, 100$$

Therefore, RR could range from 0 (*i.e.*, the two products receive the same rating) to 1 (*i.e.*, cigarettes are rated 100 and the other product is rated 0).

For categorical variables, weighted proportions and 95% CIs are provided. Statistical significance was set at *p* < 0.05 for all statistical tests, and corrections for multiple comparisons were applied when analyses of HCP specialties were conducted.

## Results

The average age of the sample was 47.1 years old, with a plurality (37.0%) of HCPs in practice for more than 20 years (see Table 1). Slightly more than half of the sample (51.8%) were female, and a similar percentage worked in a suburban location (53.8%). The vast majority of respondents (89.5%) never used any tobacco product regularly; and of those who reported any regular use, most respondents reported smoking cigarettes. Most HCPs (60.8%) either never received smoking cessation training (28.1%) or received training more than 5 years ago (32.7%).

**Table 1:** Sample demographics

	Total	NP/PA Primary Care	NP/PA Specialty	FP/GP	IM	OBGYN	CARD	PULM
	N = 700	n = 100	n = 100	n = 100	n = 100	n = 100	n = 100	n = 100
Mean Age (95% CI)	47.1 (46.0–48.2)	42.7 (40.8–44.7)	45.0 (43.0–47.0)	50.7 (48.1–53.2)	48.7 (46.2–51.1)	55.8 (53.7–57.8)	52.7 (50.3–55.2)	50.3 (48.2–52.4)
*Years in practice*								
1–5	13.7%	20.0%	15.0%	11.0%	10.0%	1.0%	6.0%	7.0%
6–10	22.5%	30.0%	24.0%	14.0%	22.0%	8.0%	17.0%	17.0%
11–15	15.7%	18.0%	18.0%	12.0%	15.0%	13.0%	13.0%	24.0%
16–20	11.2%	11.0%	11.0%	11.0%	9.0%	16.0%	17.0%	17.0%
More than 20	37.0%	21.0%	32.0%	52.0%	44.0%	62.0%	47.0%	35.0%
*Gender*								
Male	45.3%	23.0%	9.0%	59.0%	76.0%	56.0%	86.0%	68.0%
Female	51.8%	75.0%	90.0%	39.0%	18.0%	42.0%	11.0%	26.0%
Non-binary/ Prefer not to answer	2.9%	2.0%	1.0%	2.0%	6.0%	2.0%	3.0%	6.0%
*Practice location*								
Urban	31.5%	26.0%	33.0%	27.0%	41.0%	30.0%	48.0%	51.0%
Suburban	53.8%	54.0%	54.0%	52.0%	54.0%	61.0%	48.0%	44.0%
Rural	14.7%	20.0%	13.0%	21.0%	5.0%	9.0%	4.0%	5.0%
*Ever regularly used*								
Cigarettes	8.9%	11.0%	12.0%	7.0%	5.0%	13.0%	5.0%	7.0%
E-cigarettes	2.9%	4.0%	1.0%	1.0%	4.0%	3.0%	0.0%	0.0%
Smokeless Tobacco	1.6%	2.0%	2.0%	1.0%	1.0%	3.0%	1.0%	2.0%
Nicotine Pouches	1.5%	2.0%	2.0%	1.0%	1.0%	1.0%	1.0%	0.0%
None	89.5%	89.0%	87.0%	91.0%	91.0%	84.0%	93.0%	91.0%
*Most recent smoking cessation training*								
Within last year	9.6%	16.0%	4.0%	7.0%	7.0%	1.0%	4.0%	10.0%
1 − 2 years ago	11.8%	15.0%	12.0%	13.0%	8.0%	4.0%	9.0%	12.0%
3 − 5 years ago	17.7%	23.0%	14.0%	20.0%	11.0%	12.0%	12.0%	20.0%
More than 5 years ago	32.7%	23.0%	37.0%	35.0%	42.0%	43.0%	35.0%	34.0%
Never	28.1%	23.0%	33.0%	25.0%	32.0%	40.0%	40.0%	24.0%

Weighted estimates for total and unweighted estimates by specialty

*NP/PA* Nurse Practitioner/Physician Assistant, *FP/GP* Family Practice/General Practice, *IM* Internal Medicine, *OBGYN* Obstetrics and Gynecology, *CARD* Cardiology, *PULM* Pulmonology

### Health risk ratings

**Fig. 1:**
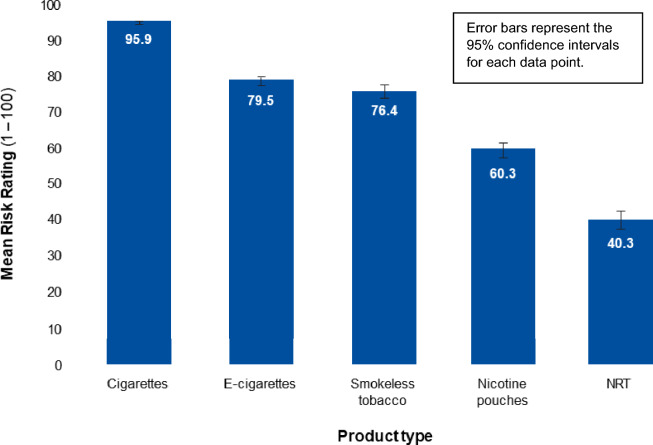
HCPs’ Mean Health Risk Ratings of Tobacco and Nicotine-Containing Products (N = 700). Weighted mean ratings and 95% CIs. Q4. “Please place each of the following products on a scale where “0” means “No Risk” and “100” means “Substantial Risk” by clicking, holding, and dragging the square in the middle of each row to the appropriate place on the scale” (Supplement questionnaire)

HCPs’ mean overall health risk ratings for cigarettes, e-cigarettes, smokeless tobacco, nicotine pouches, and NRTs are presented in Fig. 1. A one-way repeated measures ANOVA yielded a significant effect of product type (F_4,693_ = 403.9, *p* < 0.0001) on overall health risk ratings (cigarettes: 95.9 ± 0.8; e-cigarettes: 79.5 ± 1.6; smokeless tobacco: 76.4 ± 1.9; nicotine pouches: 60.3 ± 2.5; and NRTs: 40.3 ± 2.6). Post-hoc pairwise comparisons with a Tukey–Kramer adjustment for multiple comparisons indicated that the risk associated with each product is significantly different from the risk associated with every other product (all *p* < 0.001, except for e-cigarettes and smokeless tobacco for which *p* = 0.012). These findings indicate that HCPs perceive that non-combustible TNPs have less overall health risk than cigarettes. Ratings generally do not differ by HCP specialty except that the two groups of NPs/PAs perceived more risk with e-cigarettes than any of the physician specialties (see Supplemental Table 3)*.*

The overall mean perceived RRs relative to cigarettes were 17.0% for e-cigarettes, 20.2% for smokeless tobacco, 36.7% for nicotine pouches, and 57.1% for NRTs. The distributions of perceived RRs for e-cigarettes compared to cigarettes are noteworthy in that slightly more than a third (38.2%) of HCPs associated e-cigarettes with less than 10% RR relative to cigarettes and a similar number (37.0%) of HCPs associated e-cigarettes with 10–25% RR relative to cigarettes. Only about a fifth (20.2%) of HCPs associated e-cigarettes with 26–50% RR relative to cigarettes, and just 4.6*%* associated e-cigarettes with at least 51% RR relative to cigarettes (*see* Supplemental Table 4).

### Attribution of risk of lung cancer from smoking cigarettes

**Fig. 2:**
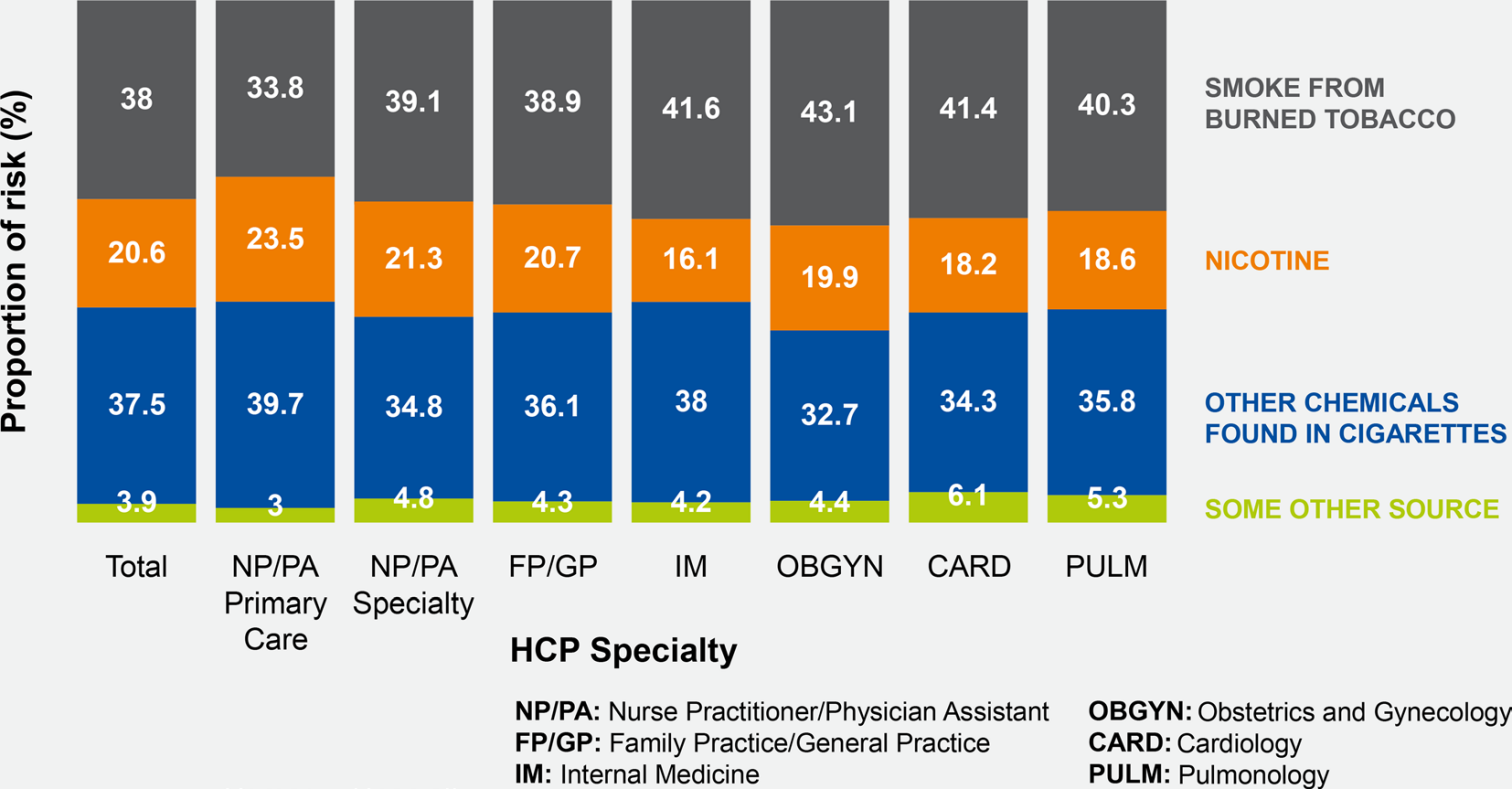
Attribution of Risk of Lung Cancer Associated with Smoking Cigarettes by HCP Specialty. Q3a. “Consider the risk of lung cancer associated with smoking cigarettes as 100%. What percent of that risk do you attribute to each of the following?” (Supplement questionnaire)

HCPs were asked to estimate the proportion of the risk of lung cancer attributable to each of the following four sources–smoke from burned tobacco, nicotine, other chemicals found in cigarettes, and some other source. Overall, HCPs attributed equal proportions of the risk of lung cancer to the smoke from burned tobacco and other chemicals in cigarettes (both ~ 38%), and about 21% of the risk to nicotine, and differences between specialties were negligible (see Fig. 2).

### Impact of method of reducing cigarette consumption on health risks

**Fig. 3:**
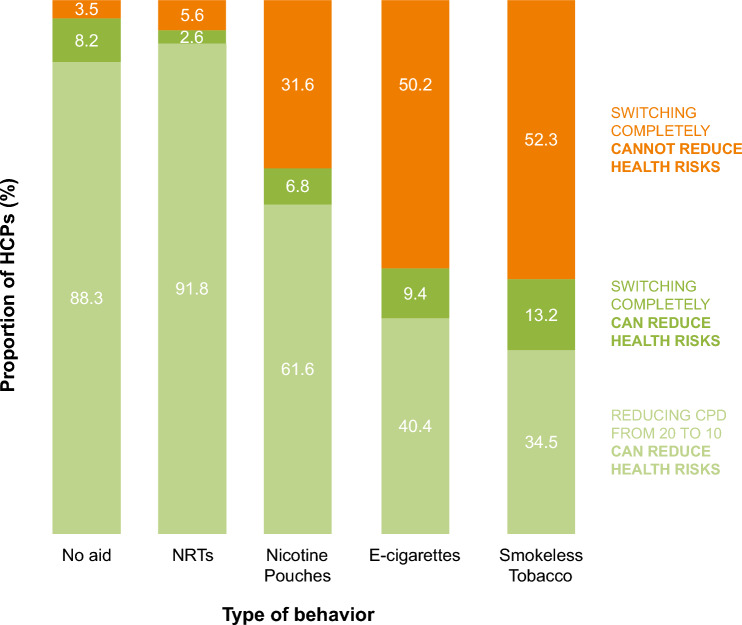
Weighted Proportion of HCPs Who Believe Reducing or Switching Completely from Cigarettes Using Different TNPs or No Product Can Reduce Health Risks from Smoking (N = 700). Q8. “Do you believe the following changes in cigarette smoking behavior can reduce the health risks of smoking?” Reducing cigarettes per day from 20 to 10…/Reducing cigarettes per day from 20 to 0… without using any other product, by using NRT, by using nicotine pouches, by using e-cigarettes, by using smokeless tobacco products (Supplement questionnaire)

HCPs were asked if they believed reducing or quitting smoking by using different methods (*i.e.*, e-cigarettes, nicotine pouches, smokeless tobacco, NRTs, or no product at all), can reduce the health risks from smoking cigarettes. Based on their answers, HCPs were placed into one of three mutually exclusive groups for each behavior: (1) reducing cigarettes per day (CPD) from 20 to 10 with the use of [product/no product] can reduce health risks; (2) quitting cigarettes (*i.e.*, reducing CPD from 20 to 0) and switching completely to [product/no product] can reduce health risks; or (3) quitting cigarettes and switching completely to [product/no product] cannot reduce health risks (see Fig. 3). A notable finding is the substantial proportion of HCPs who believe that reducing CPD from 20 to 0 *cannot* reduce health risks when using nicotine pouches (31.6%), e-cigarettes (50.2%), or smokeless tobacco (52.3%), compared to much lower percentages when using no product (4.5%) or NRT (5.6%). A chi-square test of homogeneity revealed that the proportion of HCPs selecting each outcome varies by the product used (χ^2^ = 604.2, df = 6.45, *p* < 0.05). *Post-hoc* pairwise chi-square tests of homogeneity using the Bonferroni Adjustment for multiple comparisons (*p* = 0.05/10 or 0.005) revealed significant differences between all comparisons except smokeless tobacco and e-cigarettes. Thus, for example, the distribution of responses for using *no product* to aid in reducing or switching completely (88.3% believe reducing CPD from 20 to 10 reduces health risks, 8.2% believe switching completely reduces health risks, and 3.5% do not believe switching completely reduces health risks) differed from the distribution of responses for using *nicotine pouches* (61.6%, 6.8%, and 31.6%, respectively), and both of those distributions differed from *e-cigarettes* (40.4%, 9.4%, and 50.2%, respectively).

### Univariate regressions: variables impacting belief that e-cigarettes can reduce health risks

Previous studies have shown that cigarette smokers who perceive e-cigarettes to be as harmful or more harmful than cigarettes are less likely to switch from smoking cigarettes to using e-cigarettes [12]. Thus, univariate regressions were conducted to understand which characteristics and perceptions are associated with whether HCPs believe that switching completely from cigarettes to e-cigarettes can reduce health risks (see Table 2). NP/PAs in both primary care and specialty practices were less likely than the reference group (FP/GPs) to believe that switching completely to e-cigarettes can reduce health risks (OR and 95% CIs: NP/PAs primary care: 0.55, 0.31–0.96 and NP/PAs specialty practices: 0.48, 0.27–0.85). After accounting for HCP Specialty, three other variables yielded statistically significant relationships: HCPs whose most recent smoking cessation training occurred 1–2 years ago were more likely to believe switching completely reduces health risks (1.89, 1.04–3.42); HCPs who rated the overall health risks of e-cigarettes higher were less likely to believe that switching completely reduces health risks (*p* < 0.0001); and HCPs with a higher percent RR for e-cigarettes relative to cigarettes were more likely to believe that switching completely reduces health risks (*p* < 0.0001). Regressions for smokeless tobacco and nicotine pouches show similar effects of percent RR (see Supplemental Tables 5 and 6).

**Table 2:** Univariate regression analyses regarding belief that switching completely from cigarettes to e-cigarettes reduces health risks

Independent variable	Odds Ratios (95% CI)	Parameter/ Coefficient	Standard error	Wald Chi-Square	*p* value
*HCP specialty*					
FP/GP (reference)					
NPs/PAs primary care	0.55 (0.31–0.96)	**− 0.605**	**0.286**	**4.470**	**0.03**
NPs/PAs specialty care	0.48 (0.27–0.85)	**− 0.731**	**0.288**	**6.431**	**0.01**
IM	1.18 (0.67–2.07)	0.164	0.287	0.328	0.57
OBGYN	1.04 (0.60–1.82)	0.041	0.285	0.020	0.89
CARD	1.28 (0.73–2.26)	0.248	0.288	0.743	0.39
PULM	1.28 (0.73–2.26)	0.248	0.288	0.743	0.39
*Age*		0.001	0.00676	0.007	0.01
*Gender*					
Male (reference)					
Female	0.74 (0.52–1.08)	**− **0.295	0.188	2.475	0.12
Non-binary	*Insufficient sample*				
*Tobacco history*					
None (reference)					
Used cigarettes	1.52 (0.87–2.64)	0.418	0.283	2.190	0.14
Used e-cigarettes	*Insufficient sample*				
Used smokeless tobacco	*Insufficient sample*				
Used nicotine pouches	*Insufficient sample*				
*Most recent smoking cessation training*					
None (reference)					
More than 5 years ago	0.93 (0.64–1.36)	**− **0.073	0.194	0.141	0.71
3 − 5 years ago	0.76 (0.47–1.23)	**− **0.270	0.244	1.220	0.27
1 − 2 years ago	1.89 (1.04–3.42)	**0.634**	**0.304**	**4.369**	**0.04**
Within the last year	0.64 (0.32–1.27)	**− **0.443	0.350	1.604	0.21
*Risk of lung cancer attributed to combustion*		0.000	0.004	0.005	0.94
*Risk wording:*					
Original	1.02 (0.74–1.41)	0.023	0.165	0.020	0.89
Revised (reference)					
*Overall risk ratings of:*					
Cigarettes		**− **0.016	0.010	2.676	0.10
E-cigarettes		**0.053**	**0.006**	**71.615**	** < 0.0001**
*% Risk reduction*		**4.942**	**0.591**	**69.837**	** < 0.0001**

Bold rows reflect statistically significant relationships (p < .05)

*NP/PA* Nurse Practitioner/Physician Assistant, *FP/GP* Family Practice/General Practice, *IM* Internal Medicine, *OBGYN* Obstetrics and Gynecology, *CARD* Cardiology, *PULM* Pulmonology

Q8. “Do you believe the following changes in cigarette smoking behavior can reduce the health risks of smoking?” Reducing cigarettes per day from 20 to 0… by using e-cigarettes (Supplement questionnaire)

## Discussion

In 2023, the National Institutes of Health and CTP co-launched a funding opportunity, “Public Health Communication Messaging about the Continuum of Risk for Tobacco Products,” [13] acknowledging the importance of clearly communicating about the differential risk of various TNPs. Because HCPs are a trusted source of health-related information for many, they can play an important role in educating adult patients who smoke cigarettes about TNPs. Indeed, a recent study showed that receiving information about using e-cigarettes to quit smoking from an HCP was associated with longer quit durations than when information came from other sources [14]. The same study, however, also observed that HCPs were identified as a source of advice (14%) far less often than friends (43.9%) and the internet (35.2%). Regardless, the then-Acting Director of CTP and his co-authors recently advocated for HCPs to discuss the relative risks of TNPs with adult patients who continue to smoke cigarettes [15].

Results from the current study indicate, however, that only 39.1% of HCPs have had smoking cessation training in the last five years. To the extent that smoking cessation training includes information about the relative risk of TNPs, HCPs may be relying on out-of-date information. Several findings from the current study support that possibility. First, HCPs associate e-cigarettes with approximately 17% less overall health risk than cigarettes, whereas a recent meta-analysis showed that e-cigarettes are associated with between 35 and 50% risk reduction for stroke, COPD, and MI [16]. Second, HCPs attribute only 38% of the risk of lung cancer associated with smoking cigarettes to burning tobacco, even though combustion is acknowledged to be the primary source of carcinogens in smoking [17]. The surveyed HCPs’ attribution of ~ 21% of the risk of lung cancer to nicotine is consistent with other studies that have demonstrated physicians mistakenly associate nicotine with an increased risk of cancer [8]. Third, and most importantly from a public health perspective, whereas 90% of HCPs believe that *even partial reductions* in cigarettes per day using either NRTs or no aid can reduce health risk associated with smoking cigarettes, only half of HCPs believe that *switching completely* from smoking cigarettes to e-cigarettes or smokeless tobacco can reduce those same health risks. Thus, half of the HCPs surveyed believe that switching completely from cigarettes to e-cigarettes or smokeless tobacco cannot help to reduce health risk*,* which stands in stark contrast to the substantial evidence that switching completely lowers adverse health outcomes associated with smoking (*e.g.,* cardiovascular, respiratory, oral) [3, 18].

Although it could be hypothesized that the current findings reflect HCPs’ lack of familiarity with TNPs, especially considering the absence of smoking cessation training, more than 90% of HCPs reported knowing either “a little” or “a lot” about e-cigarettes and smokeless tobacco, and 72% reported knowing either “a little” or “a lot” about nicotine pouches. Moreover, analyzing these data separately for those who know “a little” versus “a lot” reveals no difference in the proportion of HCPs who believe that switching completely can reduce health risk (*see* Supplemental Table 7). Thus, self-reported familiarity with TNPs does not appear to account for these findings, which aligns with the well-established phenomenon of a disconnect between perceived familiarity and factual knowledge [19]. By contrast, HCPs who perceive a more substantial difference in risk between cigarettes and e-cigarettes are more likely to believe that switching to e-cigarettes can reduce health risk, indicating that risk perceptions, rather than perceived familiarity, are more likely to explain the current findings.

Collectively, these findings suggest there may be challenges for HCPs to deliver accurate information about the risk continuum of TNPs when counseling adult smokers about quitting or reducing cigarette smoking. It appears, therefore, that additional education on the continuum of risk of TNPs is warranted, ideally with a comprehensive and interactive curriculum that translates to measurable patient outcomes [20], which in this instance would be quitting or switching to a reduced risk TNP. Evaluating HCPs’ perceptions over time would provide a means to ascertain if training interventions have a positive effect.

The primary limitation of the current study is that it was conducted with a convenience sample of HCPs obtained through an online panel. Although web-panel participation is a potential source of bias, demographic data suggest that the sample is broadly representative of each specialty. In addition, surveys of web-panelists are the industry standard and have been used by FDA in its own research [21–23]. A second limitation of this study is that rewording the survey question regarding the source of risk of lung cancer resulted in two statistically significant differences. The effect size of both those differences were, however, small. In addition, univariate regressions did not reveal any relationship between the wording change and believing that switching completely from cigarettes to non-combustible TNPs can reduce health risks. Thus, impact of the wording change on the overall findings was negligible. Strengths of the study are that NP/PAs were included in the sample, the overall sample was weighted to account for quota-sampling by HCP specialty, and the survey instrument was pre-tested with two respondents from each specialty.

These findings indicate that many HCPs have an inaccurate understanding of the relative risks of TNPs, which could lead to patient counseling that is inconsistent with current scientific evidence and possibly creating an obstacle for patients who want to quit smoking cigarettes. Ensuring that more HCPs have up-to-date, evidence-based information about the relative risks of TNPs would increase the likelihood that HCPs provide patients with accurate counseling regarding the continuum of risk of TNPs, which has the prospect of improving population health by helping more patients who smoke cigarettes (particularly those who have been unable to quit) to switch completely to non-combustible TNPs that potentially reduce their smoking-related health risks.

## Supplementary Information

Below is the link to the electronic supplementary material.

Supplementary file1 (DOCX 52 kb)

Supplementary file2 (DOCX 176 kb)
