# Supplementary material for: U.S. healthcare provider risk perceptions of tobacco and nicotine-containing products
Source: Intern Emerg Med. 2026 Apr 13;21(4):1325–33. doi: 10.1007/s11739-026-04321-1 (PMC13263261; doi:10.1007/s11739-026-04321-1)
Supplement: Supplementary file 1 — Supplementary file1 (DOCX 52 kb) [file 11739_2026_4321_MOESM1_ESM.docx]

**Supplemental Table 1: Attribution of Risk of Lung Cancer Associated with Smoking Cigarettes by Response Wording**

|  | **With “Inhaling” in Responses**  **(n = 384)** | **Sans “Inhaling” in Responses**  **(n = 316)** | **Unpaired *t* test and *p* value** | **Effect Size**  **(Cohen’s *d*)** |
| --- | --- | --- | --- | --- |
| [Inhaling] Smoke from burned tobacco | 36.3%  (33.9–38.7) | 41.4%  (38.2–44.6) | *t* _(698 df)_ = 2.52  *p* = 0.012 | *d* = 0.25 |
| [Inhaling] Nicotine | 22.3%  (19.9–24.8 ) | 17.2%  (14.5–19.9) | *t* _(698 df)_ = 2.73 *p* = 0.006 | *d* = 0.27 |
| [Inhaling] Other chemicals found in cigarettes | 37.5%  (34.9–40.1) | 37.5%  (34.3–40.6) | *t* _(698 df)_ = 0.01  *p* = 0.992 | *d* = 0.001 |
| Some other source | 3.9%  (3.1–4.7) | 3.9%  (3.0–4.8) | *t* _(698 df)_ = 0.02  *p* = 0.982 | *d* = 0.002 |

Weighted proportions and 95% CIs.

Q3a. “Consider the risk of **lung cancer** associated with smoking cigarettes as 100%. What percent of that risk do you attribute to each of the following?”

**Supplemental Table 2: Counts and Weights by HCP Specialty**

|  | **NP/PA**  **Primary Care** | **NP/PA**  **Specialty** | **FP/GP** | **IM** | **OBGYN** | **CARD** | **PULM** |
| --- | --- | --- | --- | --- | --- | --- | --- |
| Universe | 237,404 | 183,520 | 122,005 | 138,048 | 43,678 | 24,079 | 4,206 |
| Eligibility Rate | 95% | 30%* | 92% | 97% | 95% | 91% | 97% |
| Eligible Universe | 226,467 | 55,493 | 112,551 | 133,971 | 41,406 | 21,811 | 4,063 |
| Sample Size | 100 | 100 | 100 | 100 | 100 | 100 | 100 |
| Weight | 2264.67 | 554.93 | 1125.51 | 1339.71 | 414.06 | 218.11 | 40.63 |

NP/PA: Nurse Practitioner/Physician Assistant; FP/GP: Family Practice/General Practice; IM: Internal Medicine; OBGYN: Obstetrics and Gynecology; CARD: Cardiology; PULM: Pulmonology

* The lower eligibility rate among NP/PAs in Specialty practices reflects ineligibility of practitioners in specialties other than obstetrics/gynecology, cardiology, and pulmonology.

**Supplemental Table 3: Overall Risk Ratings by HCP Specialty**

|  | **NP/PA**  **Primary Care** | **NP/PA**  **Specialty** | **FP/GP** | **IM** | **OBGYN** | **CARD** | **PULM** |
| --- | --- | --- | --- | --- | --- | --- | --- |
| Cigarettes | 96.4  (94.6–98.1) | 96.6  (94.8–98.3) | 94.8  (92.9–96.7) | 96.0  (94.8–97.1) | 96.3  (95.0–97.6) | 94.5  (93.0–96.1) | 94.5  (92.9–96.0) |
| E-cigarettes | 83.7  (80.8–86.5) | 83.8  (80.3–87.4) | 76.0  (71.7–80.2) | 74.7  (71.3–78.2) | 78.9  (75.2–82.6) | 76.6  (73.2–80.0) | 72.1  (68.2–76.0) |
| Smokeless Tobacco | 76.0  (72.2–79.8) | 79.3  (76.0–82.6) | 75.5  (71.3–79.7) | 77.3  (74.1–80.6) | 75.2  (71.3–79.1) | 74.7  (71.3–78.0) | 74.5  (71.3–77.7) |
| Nicotine Pouches | 61.9  (57.0–66.7) | 63.2  (58.3–68.1) | 62.7  (57.4–67.9) | 54.8  (49.6–59.9) | 60.0  (54.9–65.1) | 59.7  (55.1–64.4) | 56.3  (51.1–61.4) |
| NRTs | 39.9  (34.4–45.3) | 41.1  (36.4–45.8) | 43.0  (37.9–48.1) | 38.4  (33.2–43.7) | 41.1  (36.0–46.2) | 41.0  (35.7–46.3) | 36.0  (30.4–41.5) |

Unweighted mean ratings and 95% CIs.

NP/PA: Nurse Practitioner/Physician Assistant; FP/GP: Family Practice/General Practice; IM: Internal Medicine; OBGYN: Obstetrics and Gynecology; CARD: Cardiology; PULM: Pulmonology

Q4. “Please place each of the following products on a scale where “0” means “No Risk” and “100” means “Substantial Risk” by clicking, holding, and dragging the square in the middle of each row to the appropriate place on the scale.”

**Supplemental Table 4: Weighted Proportion of HCPs Attributing Each Level of Perceived Risk Reduction for E-Cigarettes Relative to Cigarettes**

|  | **Percent Risk Reduction* Compared to Cigarettes for:** | | | |
| --- | --- | --- | --- | --- |
|  | **E-Cigarettes** | **Smokeless Tobacco** | **Nicotine Pouches** | **NRTs** |
| Less than 10% | 38.2%  (31.4–44.9) | 30.7%  (24.8–36.6) | 17.1%  (12.9–21.4) | 7.5%  (4.6–10.3) |
| 10% – 25% | 37.0%  (30.2–43.8) | 38.0%  (31.3–44.7) | 22.0%  (17.0–26.9) | 10.1%  (6.9–13.2) |
| 26% – 50% | 20.2%  (15.6–24.9) | 23.0%  (18.1–28.0) | 33.4%  (27.2–39.7) | 23.0%  (18.2–27.8) |
| 51% – 75% | 3.4%  (1.9–4.8) | 7.0%  (4.2–9.8) | 15.3%  (11.4–19.2) | 24.2%  (19.0–29.4) |
| 76% or more | 1.2%  (0.3–2.1) | 1.3%  (0.2–2.3) | 12.2%  (8.7–15.7) | 35.3%  (28.8–41.8) |
| **MEAN RISK REDUCTION** | **17.0%**  **(15.4–18.6)** | **20.2%**  **(18.3–22.0)** | **36.7%**  **(34.1–39.3)** | **57.1%**  **(54.2–60.0)** |

Weighted proportions and 95% CIs.

*RR_e-cig_ = (Rating_cig_ – Rating_e-cig_) / Rating_cig_

**Supplemental Table 5: Univariate Regression Analyses Regarding Belief that Switching Completely from Cigarettes to Smokeless Tobacco Reduces Health Risks**

| **Independent Variable** | Parameter/  Coefficient | Standard Error | Wald  Chi-Square | *p* value |  | |
| --- | --- | --- | --- | --- | --- | --- |
| *HCP Specialty* | | | | | |  |
| FP/GP (reference) |  |  |  |  |  | |
| NPs/PAs Primary Care | -0.4418 | 0.2846 | 2.4104 | 0.12 | |  |
| NPs/PAs Specialty Care | -0.4013 | 0.2843 | 1.9933 | 0.16 | |  |
| IM | -0.2007 | 0.2836 | 0.5008 | 0.48 | |  |
| OBGYN | -0.4013 | 0.2843 | 1.9933 | 0.16 | |  |
| CARD | -0.3208 | 0.2838 | 1.2778 | 0.26 | |  |
| PULM | -0.1607 | 0.2836 | 0.3210 | 0.57 | |  |
| *Age* | -0.00784 | 0.00666 | 1.3847 | 0.24 | |  |
| *Gender* | | | | | |  |
| Male (reference) |  |  |  |  |  | |
| **Female** | **-0.4243** | **0.1872** | **5.1377** | **0.02** | |  |
| Non-binary | *Insufficient sample* | | | | |  |
| *Tobacco History* | | | | | |  |
| None (reference) |  |  |  |  |  | |
| Used cigarettes | 0.3651 | 0.2743 | 1.772 | 0.18 | |  |
| Used e-cigarettes | *Insufficient sample* | | | | |  |
| Used smokeless tobacco | *Insufficient sample* | | | | |  |
| Used nicotine pouches | *Insufficient sample* | | | | |  |
| *Most Recent Smoking Cessation Training* | | | | | |  |
| None (reference) |  |  |  |  |  | |
| More than 5 years ago | -0.073 | 0.1944 | 0.1412 | 0.70 | |  |
| 3−5 years ago | -0.1041 | 0.2436 | 0.1825 | 0.67 | |  |
| 1−2 years ago | 0.532 | 0.2824 | 3.5489 | 0.06 | |  |
| Within the last year | 0.477 | 0.3385 | 1.9863 | 0.16 | |  |
| *Risk of Lung Cancer Attributed to Combustion* | 0.0018 | 0.0036 | 0.2555 | 0.61 | |  |
| *Risk Wording:* | | | | | |  |
| Original | 0.1578 | 0.1621 | 0.9483 | 0.33 | |  |
| Revised (reference) |  |  |  |  | |  |
| *Overall Risk Ratings of:* | | | | | |  |
| **Cigarettes** | **-0.0293** | **0.01** | **8.5534** | **0.003** | |  |
| **Smokeless Tobacco** | **-0.0441** | **0.0051** | **73.6544** | **<0.001** | |  |
| ***% Risk Reduction*** | **3.8364** | **0.4944** | **60.2045** | **<0.001** | |  |

Bold rows reflect statistically significant relationships.

NP/PA: Nurse Practitioner/Physician Assistant; FP/GP: Family Practice/General Practice; IM: Internal Medicine; OBGYN: Obstetrics and Gynecology; CARD: Cardiology; PULM: Pulmonology

Q8. “Do you believe the following changes in cigarette smoking behavior can reduce the health risks of smoking?”

Reducing cigarettes per day from 20 to 0… by using nicotine pouches.

**Supplemental Table 6: Univariate Regression Analyses Regarding Belief that Switching Completely from Cigarettes to Nicotine Pouches Reduces Health Risks**

| **Independent Variable** | Parameter/  Coefficient | Standard Error | Wald  Chi-Square | *p* value |  | |
| --- | --- | --- | --- | --- | --- | --- |
| *HCP Specialty* | | | | | |  |
| FP/GP (reference) |  |  |  |  |  | |
| NPs/PAs Primary Care | -0.1907 | 0.3091 | 0.3805 | 0.54 | |  |
| NPs/PAs Specialty Care | -0.4123 | 0.3041 | 1.8373 | 0.18 | |  |
| IM | -0.1907 | 0.3091 | 0.3805 | 0.54 | |  |
| OBGYN | -0.1443 | 0.3104 | 0.2162 | 0.64 | |  |
| CARD | -0.1443 | 0.3104 | 0.2162 | 0.64 | |  |
| PULM | 0.0502 | 0.3168 | 0.0251 | 0.87 | |  |
| *Age* | -0.0074 | 0.0071 | 1.0834 | 0.30 | |  |
| *Gender* | | | | | |  |
| Male (reference) |  |  |  |  |  | |
| **Female** | **-0.5643** | **0.2019** | **7.8125** | **0.005** | |  |
| Non-binary | *Insufficient sample* | | | | |  |
| *Tobacco History* | | | | | |  |
| None (reference) |  |  |  |  |  | |
| Used cigarettes | 0.1009 | 0.2971 | 0.1152 | 0.73 | |  |
| Used e-cigarettes | *Insufficient sample* | | | | |  |
| Used smokeless tobacco | *Insufficient sample* | | | | |  |
| Used nicotine pouches | *Insufficient sample* | | | | |  |
| *Most Recent Smoking Cessation Training* | | | | | |  |
| None (reference) |  |  |  |  |  | |
| More than 5 years ago | -0.0573 | 0.2039 | 0.0791 | 0.78 | |  |
| 3−5 years ago | -0.4262 | 0.2543 | 2.8102 | 0.09 | |  |
| **1−2 years ago** | 0.1555 | 0.3176 | 0.2398 | 0.62 | |  |
| Within the last year | -0.1087 | 0.3594 | 0.0915 | 0.76 | |  |
| *Risk of Lung Cancer Attributed to Combustion* | 0.0011 | 0.0039 | 0.0808 | 0.78 | |  |
| *Risk Wording:* | | | | | |  |
| Original | 0.0661 | 0.1744 | 0.1434 | 0.70 | |  |
| Revised (reference) |  |  |  |  | |  |
| *Overall Risk Ratings of:* | | | | | |  |
| Cigarettes | -0.0208 | 0.0114 | 3.3631 | 0.07 | |  |
| **Nicotine Pouches** | **-0.0425** | **0.0044** | **92.0247** | **<0.001** | |  |
| ***% Risk Reduction*** | **3.5746** | **0.3988** | **80.3566** | **<0.001** | |  |

Bold rows reflect statistically significant relationships.

NP/PA: Nurse Practitioner/Physician Assistant; FP/GP: Family Practice/General Practice; IM: Internal Medicine; OBGYN: Obstetrics and Gynecology; CARD: Cardiology; PULM: Pulmonology

Q8. “Do you believe the following changes in cigarette smoking behavior can reduce the health risks of smoking?”

Reducing cigarettes per day from 20 to 0… by using nicotine pouches.

**Supplemental Table 7: Weighted Proportions of HCPs Who Believe Switching Completely from Cigarettes to E-cigarettes/Smokeless Tobacco/Nicotine Pouches Can Reduce Health Risks, by Self-Reported Knowledge of Each Respective Type of Product**

|  | **E-cigarettes** | | **Smokeless**  **Tobacco** | | **Nicotine**  **Pouches** | |
| --- | --- | --- | --- | --- | --- | --- |
|  | **Know**  **a little** | **Know**  **a lot** | **Know**  **a little** | **Know**  **a lot** | **Know**  **a little** | **Know**  **a lot** |
| n = | 175 | 190 | 162 | 155 | 201 | 146 |
| % believe switching completely from cigarettes to e-cigarettes / smokeless tobacco / nicotine pouches can reduce health risks | 51.6%  (44.5–58.7) | 47.8%  (41.0–54.5) | 50.2%  (43.1–57.3 | 46.6%  (39.7–53.5) | 71.9%  (65.4–78.4 | 76.1%  (68.3–83.9) |

Weighted proportions and 95% CIs.
