# Supplementary material for: U.S. healthcare provider risk perceptions of tobacco and nicotine-containing products
Source: Intern Emerg Med. 2026 Apr 13;21(4):1325–33. doi: 10.1007/s11739-026-04321-1 (PMC13263261; doi:10.1007/s11739-026-04321-1)
Supplement: Supplementary file 2 — Supplementary file2 (DOCX 176 kb) [file 11739_2026_4321_MOESM2_ESM.docx]

**Supplemental Material: Screener and Relevant Survey Questions**

**Screener**

S1. Which of the following United States medical licenses do you currently hold?

| **Select One** |  |
| --- | --- |
| 🔾 | MD/DO |
| 🔾 | Nurse Practitioner |
| 🔾 | Physician Assistant |
| 🔾 | Other |
| 🔾 | None of the above |

**PROGRAMMER:**

1. **CLASSIFY SPECIALTIES AS FOLLOWS FOR QUOTAS:**
   1. **MD/DO = ROW 1**
   2. **NP/PA = ROW 2 OR 3**
2. **IF ROW 4 “OTHER” or ROW 5 “NONE OF THE ABOVE”, TERMINATE NOW**

S2. What is the **primary** area in which you practice?

| **Select One** |  |
| --- | --- |
| 🔾 | Allergy/Immunology |
| 🔾 | Cardiology |
| 🔾 | Emergency Medicine |
| 🔾 | Family Medicine |
| 🔾 | General Surgery |
| 🔾 | Internal Medicine |
| 🔾 | Obstetrics/Gynecology |
| 🔾 | Oncology |
| 🔾 | Otolaryngology |
| 🔾 | Pediatrics |
| 🔾 | Pulmonology |
| 🔾 | Urology |
| 🔾 | Other (Please specify) |

**PROGRAMMER:**

1. **ASK ALL**
2. **IF ROW 1, 3, 5, 8, 9, 10, 12, OR 13 IS SELECTED, TERMINATE NOW**

S3. In what state do you primarily practice?

**[SHOW DROP-DOWN LIST OF STATES]**

**PROGRAMMER: TERMINATE NOW IF ME, MN, OR VT**

S4. What proportion of your week do you typically spend in in-person or virtual direct patient care or office consultations (rather than in medical procedures, the operating room, doing research, administrative tasks, etc.)?

**Percent of Week Typically Spent in Direct Patient Care:**

**_____________%**

**PROGRAMMER:**

1. **RANGE 0**−**100%**
2. **TERMINATE IF < 50%**

**INFORMED CONSENT STATEMENT**

**Protocol No.:** S&RA-BR-2023-12

**Investigator:** Michael Polster, PhD

1835 Market Street

25^th^ floor

Philadelphia, Pennsylvania 19103

United States

**STUDY-RELATED**

**PHONE NUMBER(S):** Robyn Chae

215-496-6941 (24 hours)

rchae@naxionthinking.com

You are being invited to take part in a survey. Taking part in this survey is voluntary. Whether you participate is your decision. You can choose not to participate now or agree to participate and later change your mind. There will be no penalty or loss of benefits to which you are otherwise entitled.

If you participate in this survey, you will be responsible for answering questions as honestly as you can.

Participation will not cost you anything other than about 15 minutes of your time. You will receive an incentive of $**[**INSERT INCENTIVE**]** for your participation. There are no other benefits to you for participating in this survey.

There is the potential for the risk of a loss of confidentiality of your research-related information. Any information you provide that may be able to identify you will be kept confidential. We protect your personally identifiable information from disclosure to others to the extent required by law. We cannot promise complete secrecy. The findings from this survey may be published, but all findings will be reported collectively and not at the individual respondent level.

The answers to the surveys may be shared with individuals and organizations, including the survey sponsor, individuals who work with the sponsor, and the Institutional Review Board (IRB) that reviewed and oversees the conduct of this survey.

If you have questions, concerns, or complaints, or think this survey has harmed you in any way, contact the research team at 215-496-6941 (24 hours) or rchae@naxionthinking.com.

This survey is being overseen by an Institutional Review Board (“IRB”), which is a group of people who perform independent review of research studies. You may talk to them at 855-818-2289 or researchquestions@wcgirb.com if:

You have questions, concerns, or complaints that are not being answered by the survey team or you have questions about your rights as a survey participant.

You are not getting answers from the survey team or cannot reach the survey team.

You want to talk to someone else about the survey.

By proceeding to the next screen, you confirm that you have read, understand, and accept the points above and are consenting to participate in this survey.

1. Yes, I agree **[CONTINUE]**
2. No, I do not agree **[SEND TO NON-AGREEMENT SCREEN]**

_____________________________________________________________________________________

**Survey Questions Referenced in This Report**

Q1. How familiar are you with the following types of products?

| **Cigarettes**  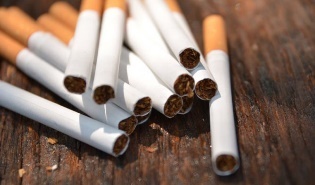 | [**DROP DOWN** – I have never heard of them, I have heard of them but do not know anything about them, I know a little about them, I know a lot about them] |
| --- | --- |
| **E-cigarettes**  These are battery-powered devices that produce an aerosol containing nicotine.  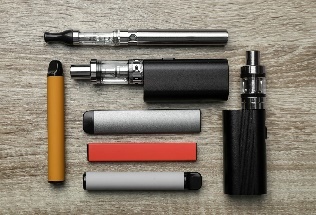 | [**DROP DOWN** – I have never heard of them, I have heard of them but do not know anything about them, I know a little about them, I know a lot about them] |
| **Smokeless tobacco products**  These include chewing tobacco, moist snuff, and snus pouches.  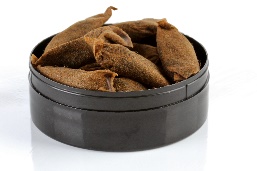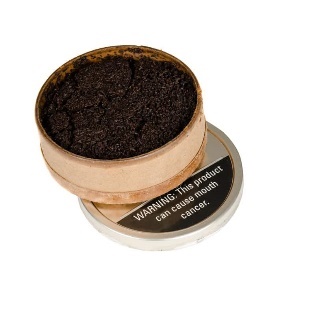 | [**DROP DOWN** – I have never heard of them, I have heard of them but do not know anything about them, I know a little about them, I know a lot about them] |
| **Nicotine pouches**  These are small fleece pouches that contain nicotine (but not tobacco) and are placed in the mouth between the lip and gum. These are not nicotine replacement therapy (NRT) products.  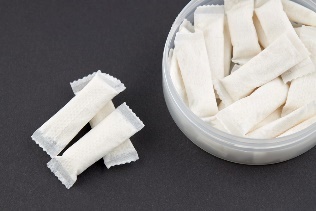 | [**DROP DOWN** – I have never heard of them, I have heard of them but do not know anything about them, I know a little about them, I know a lot about them] |
| **Nicotine replacement therapy (NRT).**  Products that provide low doses of nicotine to help people to quit smoking. Available in gum, patch, nasal spray, inhaler, and lozenge forms.  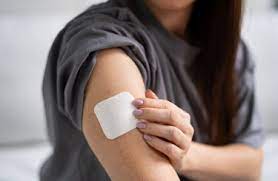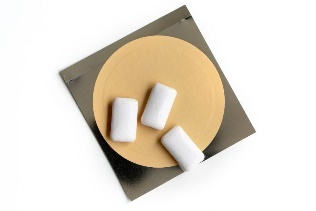 | [**DROP DOWN** – I have never heard of them, I have heard of them but do not know anything about them, I know a little about them, I know a lot about them] |

Q3a. Consider the risk of **lung cancer** associated with smoking cigarettes as 100%. What percent of that risk do you attribute to each of the following? *The total must sum to 100%.*

|  | **% of risk** |
| --- | --- |
| Inhaling smoke from burned tobacco | **____%** |
| Inhaling nicotine | **____%** |
| Inhaling other chemicals found in cigarettes | **____%** |
| Some other source | **____%** |
| **Total =** | **100%** |

**PROGRAMMER:**

1. **SET CELL RANGE 0**−**100**
2. **TOTAL MUST SUM TO 100**
3. **RANDOMIZE ORDER OF FIRST TWO ROWS**

__________________________________________________________________________________

Q4. Now we want to understand your perceptions of the ***overall*** health-related risks of different types of products. Please place each of the following products on a scale where “0” means “No Risk” and “100” means “Substantial Risk” by clicking, holding, and dragging the square in the middle of each row to the appropriate place on the scale.

|  | No Risk  ⇩ | | |  | | | | | Substantial Risk  ⇩ | | |
| --- | --- | --- | --- | --- | --- | --- | --- | --- | --- | --- | --- |
|  | 0 | 10 | 20 | 30 | 40 | 50 | 60 | 70 | 80 | 90 | 100 |
| **Cigarettes** |  |  |  |  |  |  |  |  |  |  |  |
| **E-cigarettes** |  |  |  |  |  |  |  |  |  |  |  |
| **Smokeless tobacco** |  |  |  |  |  |  |  |  |  |  |  |
| **Nicotine pouches** |  |  |  |  |  |  |  |  |  |  |  |
| **Nicotine replacement therapy** (NRT) |  |  |  |  |  |  |  |  |  |  |  |

**PROGRAMMER:**

1. **USE SLIDERS STARTING IN THE MIDDLE OF THE SCALE (AT 50)**
2. **INCLUDE HANDLE ON SLIDERS**
3. **INCLUDE VALUE ON SLIDERS**
4. **IF THE RESPONSE FOR ANY ROW EQUALS 50, THEN SHOW THIS WARNING MESSAGE:**

**You did not alter the original position of the cursor on the sliding scale for these products: [INSERT PRODUCTS FOR WHICH RESPONSE EQUALS 50]. If that was your intention, click ''OK to Continue?'' below and then click Forward. Otherwise, revise your response below and then click Forward.**

____________________________________________________________________________________

Q8. Do you believe the following changes in cigarette smoking behavior can reduce the health risks of smoking?

|  | **Reduces Health Risk** | |
| --- | --- | --- |
|  | **Yes** | **No** |
| **Reducing cigarettes per day from 20 to 10 …** | | |
| without using any other product | 🔾 | 🔾 |
| by using e-cigarettes | 🔾 | 🔾 |
| by using nicotine pouches | 🔾 | 🔾 |
| by using smokeless tobacco products | 🔾 | 🔾 |
| by using nicotine replacement therapy (NRT) | 🔾 | 🔾 |
| **Reducing cigarettes per day from 20 to 0 …** | | |
| without using any other product | 🔾 | 🔾 |
| by switching completely to e-cigarettes | 🔾 | 🔾 |
| by switching completely to nicotine pouches | 🔾 | 🔾 |
| by switching completely to smokeless tobacco products | 🔾 | 🔾 |
| by switching completely to nicotine replacement therapy (NRT) | 🔾 | 🔾 |

**PROGRAMMER:**

**1. SHOW EACH SUBSEQUENT ROW ONLY WHEN PRECEDING ROWS HAVE BEEN ANSWERED**

**2. SHOW ROW IN SECOND SET ONLY IF CORRESPONDING ROW IN THE FIRST SET IS “NO”**

These last few questions are for classification purposes only.

Q12. How old are you?

**______** Years

**PROGRAMMER: RANGE IS 18–99**

Q13. What is your gender?

|  | **Select One** |
| --- | --- |
| Male | 🔾 |
| Female | 🔾 |
| Non-binary | 🔾 |
| Prefer not to answer | 🔾 |

_____________________________________________________________________________________

Q14. For how long have you been [practicing medicine/practicing as a licensed nurse practitioner/practicing as a licensed physician assistant]?

|  | **Select One** |
| --- | --- |
| Less than 1 year | 🔾 |
| 1−5 years | 🔾 |
| 6−10 years | 🔾 |
| 11−15 years | 🔾 |
| 16−20 years | 🔾 |
| More than 20 years | 🔾 |

**PROGRAMMER**:

1. **INSERT FIRST TEXT IN BRACKETS FOR MD/DO (S1 R1)**
2. **INSERT SECOND TEXT IN BRACKETS FOR NP (S1 R2)**
3. **INSERT THIRD TEXT IN BRACKETS FOR PA (S1 R3)**

Q15. When did you most recently receive training or attend a course about strategies to help patients stop smoking cigarettes?

|  | **Select One** |
| --- | --- |
| I have never received training or attended a course | 🔾 |
| More than 5 years ago | 🔾 |
| 3−5 years ago | 🔾 |
| 1−2 years ago | 🔾 |
| Within the last year | 🔾 |

**_____________________________________________________________________________________**

Q16. Would you say the area in which you primarily practice is:

| 🔾 | Urban |
| --- | --- |
| 🔾 | Suburban |
| 🔾 | Rural |

Q17a. Which of the following products have you ever used regularly? Select all that apply.

| Cigarettes | □ |
| --- | --- |
| E-cigarettes | □ |
| Smokeless tobacco | □ |
| Nicotine pouches | □ |
| None of the above | 🔾 |

Q17b. Have you ever used nicotine replacement therapy (such as gum, lozenge, or patch) to try to quit smoking?

|  | **Select One** |
| --- | --- |
| Yes | 🔾 |
| No | 🔾 |

**PROGRAMMER: ASK IF Q17a “CIGARETTES” IS SELECTED**
